# Brevilin A is a potent anti-metastatic CRC agent that targets the VEGF-IL6-STAT3 axis in the HSCs-CRC interplay

**DOI:** 10.1186/s12967-023-04087-6

**Published:** 2023-04-16

**Authors:** Xueying Fan, Mingjing Meng, Baoting Li, Hui Chen, Jincheng Tan, Keyang Xu, Shilin Xiao, Hiu-Yee Kwan, Zhongqiu Liu, Tao Su

**Affiliations:** 1 Guangdong Provincial Key Laboratory of Translational Cancer Research of Chinese Medicines, Joint International Research Laboratory of Translational Cancer Research of Chinese Medicines, International Institute for Translational Chinese Medicine, School of Pharmaceutical Sciences, Guangzhou University of Chinese Medicine, Guangzhou, 510006 Guangdong China; 2 Centre for Cancer & Inflammation Research, School of Chinese Medicine, Hong Kong Baptist University, Hong Kong, China; 3 State Key Laboratory of Quality Research in Chinese Medicine, Macau University of Science and Technology, Macao, China; 4 Guangdong-Hong Kong-Macau Joint Lab on Chinese Medicine and Immune Disease Research, Guangzhou University of Chinese Medicine, Guangzhou, Guangdong China

**Keywords:** Brevilin A, Liver metastases of colorectal cancer, HSCs-CRC interplay, Carcinoma-associated fibroblasts, VEGF-IL6-STAT3 axis

## Abstract

**Background:**

More than half of the colorectal cancer (CRC) patients will develop liver metastasis that underlies the cancer mortality. In the hepatic tumor microenvironment, the interplay between CRC cells and hepatic stellate cells (HSCs), and the activation of HSCs to become carcinoma-associated fibroblasts (CAFs) will further promote the cancer development. Nevertheless, the critical signaling molecule that involved in these processes remains unknown, which hinders the development of effective therapeutic agents for the treatment of metastatic CRC (mCRC).

**Methods:**

Conditioned medium system and co-cultured system were used to examine the interplay between CRC cells and HSCs. Luminex liquid suspension chip detection and enzyme-linked immunosorbent assay were used to screen for the mediators in the conditioned medium that facilitated the CRC-HSCs interplay and HSCs-to-CAFs differentiation. Cell and animal models were used to examine whether brevilin A inhibited CRC liver metastasis via the VEGF-IL6-STAT3 axis.

**Results:**

In the CRC-HSCs interplay, CRC promoted HSCs-to-CAFs differentiation by releasing vascular endothelial growth factor (VEGF); and HSCs released interleukin 6 (IL6) that activated signal transducer and activator of transcription 3 (STAT3) in the CRC and hence increased the cancer metastatic potential. The functions of the VEGF-IL6-STAT3 axis in the HSCs-CRC interplay were further validated by VEGF recombinant protein and IL6 neutralizing antibody. More importantly, brevilin A, an active compound isolated from *Centipeda minima* (L.) A. Br. et Aschers, targeted the VEGF-IL6-STAT3 axis in the CRC-HSCs interplay, hence significantly inhibited colorectal liver metastasis and cancer growth both in vitro and in vivo.

**Conclusions:**

We are the first to demonstrate brevilin A possesses potent anti-mCRC effect by targeting the VEGF-IL6-STAT3 axis in the CRC-HSCs interplay. Our findings not only support the development of brevilin A as a novel therapeutic agent for mCRC treatment, but also pave the path for the development of other VEGF-IL6-STAT3 targeting therapeutic strategies.

**Supplementary Information:**

The online version contains supplementary material available at 10.1186/s12967-023-04087-6.

## Background

Paget’s “seed and soil” hypothesis and Ewing’s Theory hypothesized that formation of premetastatic niche and mechanical factors are the critical determinants for cancer organ-specific metastasis [1]. With the hemodynamic features and the specific location that allows blood flow in the sinusoidal capillaries, liver has become a unique organ to arrest circulating cancer cells. Indeed, in colorectal cancer (CRC), approximately 50% of the patients develop liver metastases, which underlies the cancer mortality [2, 3].

Cancer metastasis is a multi-step process initiated from the invasion of local cancer cells, entry of the cancer cells into the vasculature and colonization at distal sites and organs such as liver [4]. Furthermore, after reaching the liver, cancer cells will encounter different cell types in the tumor microenvironment (TME) such as parenchymal hepatocytes and nonparenchymal hepatocytes including hepatic stellate cells (HSCs), liver sinusoidal endothelial cells, Kupffer cells, dendritic cells and liver-associated lymphocytes [5]. Regarding CRC, the interactions between CRC cells and these various cell types in the TME are the critical step to control the cancer development. Indeed, many studies have suggested that the metastatic potential of CRC not only depends on pro-metastatic oncogenes, but also the tumor–stroma interaction within the TME that facilitates the cancer metastasis [6].

The carcinoma-associated fibroblasts (CAFs) are the main component in TME, which consist of stromal fibroblasts and α-smooth muscle actin (α-SMA)-positive myofibroblasts [7, 8]. In colorectal liver metastasis, the interactions between CRC and CAFs activate many signaling pathways that promote the cancer development [9, 10]. For example, CAFs release stromal cell-derived factor 1 (SDF-1) to promote colorectal liver metastasis [11]; while CRC with elevated transforming growth factor beta-1 (TGF-β1) expression also induces CAFs to synthesis extracellular matrix (ECM) constituents such as fibronectin and collagen I that promote the cancer metastasis and growth [12–14]. CAFs could arise from different origins including fibroblasts, preadipocytes, smooth muscle cells, or bone marrow-derived progenitor cells [15]. In the livers, CAFs could arise from activated HSCs. Treating quiescent HSCs with TGF-β1 induces HSCs-to-CAFs differentiation in vitro [9, 12]; and blocking TGF-β1 action abolishes the differentiation and inhibits colorectal liver metastasis [12]. Given the importance of TGF-β1 in HSCs-to-CAFs differentiation, TGF-β1 targeted therapy may be a choice to treat metastatic CRC (mCRC). However, clinical trials testing TGF inhibitors with refractory CRC showed that the overall response rate only reached 3.4% [16] that was far from satisfactory. The results in the clinical trials also imply that HSCs-to-CAFs differentiation within TME is a complicated process, it may involve other HSC intracellular factors and paracrine stimuli from the cancer cells [17]. Therefore, TGF-β1 may not be the critical regulatory factor for the HSCs-to-CAFs differentiation. Identifying the critical mediator that mediates HSCs-to-CAFs differentiation and promotes CRC metastasis and growth can provide us a comprehensive picture of the interplay between HSCs, CAFs and CRC cells, that will facilitate the development of novel therapeutic strategy to treat mCRC.

Brevilin A is a sesquiterpene lactone isolated from *Centipeda minima* (L.) A. Br. et Aschers [18]. Brevilin A exhibits anti-cancer effects in various cancers including CRC [19], it induces apoptosis and autophagy via the mitochondrial pathway and PI3K/AKT/mTOR inactivation [19]. Brevilin A also reverses the vincristine resistance of CRC by down-regulating multidrug resistance protein-1 expression [20]. However, whether brevilin A interrupts the interplay between CRC and HSCs-to-CAFs differentiation is not known.

In this study, we have identified the role of VEGF-IL6-STAT3 axis in the HSCs-to-CAFs differentiation that promotes CRC metastasis and growth. Furthermore, we found that brevilin A exhibits a potent anti-mCRC effect by targeting the VEGF-IL6-STAT3 axis. Our study provides solid scientific evidence to support the clinical translation of brevilin A into an effective therapeutic agent for mCRC treatment.

## Materials and methods

### Reagents and antibodies

Antibodies against α-SMA (Santa Cruz, Cat# SC-53142), GAPDH (Santa Cruz, Cat# SC-32233), VEGF (Santa Cruz, Cat# SC-7269) and MMP2 (Santa Cruz, Cat# SC-13595) were purchased from Santa Cruz Biotechnology (Santa Cruz, CA, USA), antibodies against phospho-STAT3 (Tyr705) (CST, Cat# 9145S), STAT3 (CST, Cat# 9139), phospho-Src (Tyr416) (CST, Cat# 6943), SRC (CST, Cat# 2109), phospho-JAK2 (Tyr1007/1008) (CST, Cat# 3771), JAK2 (CST, Cat# 3230), Cleaved-caspase 3 (CST, Cat# 9664) and goat anti rabbit HRP (CST, Cat# 7074) were purchased from Cell Signaling Technology (CST, MA, USA), antibody against Ki67 (Abcam, Cat# ab16667) was purchased from Abcam plc. (Abcam, Cambridge, UK), antibody against α-SMA (Proteintech, Cat# 14395-1-AP) was purchased from Proteintech Group, Inc. (Proteintech, CHI, USA), antibody against FAP (ABclonal, Cat# A11572) was purchased from ABclonal Technology Co., Ltd. (ABclonal, Wuhan, China), goat anti mouse IgG HRP (Mu Biotech, Cat# 125035) was purchased from Mu Biotechnology, Inc. (Mu Biotech, Guangzhou, China). Multimer anti rabbit/mouse IgG HRP for IHC was purchased from Wuhan BOSTER Bioengineering Co., Ltd (BPSTER, Wuhan, China). Protein markers were supplied by Vazyme Biotech Co., Ltd. (Vazyme, Nanjing, China). Recombinant human VEGF_165_(VEGFA) was purchased from PeproTech Inc. (PeproTech, NJ, USA). IL6 neutralizing antibody was purchased from Sino Biological, Inc. (Sino Biological, Beijing, China). VEGF165/121 neutralizing antibody was purchased from Proteintech Group, Inc. (Proteintech, Wuhan, China). Brevilin A (purity > 98%) was purchased from Chengdu Desite Biotechnology Co. Ltd. (Sichuan Provience, China). BCA protein assay reagents were purchased from Thermo Fisher Scientific (Thermor Fisher, MA, USA). 3-(4,5-Dimethylthiazol-2-yl)-2,5-diphenyltetrazolium bromide (MTT) and dimethyl sulfoxide (DMSO) were purchased from Sigma Chemicals Ltd. (St. Louis, MO, USA). IL6, IL8 and VEGF ELISA commercial kits were purchased from Quanzhou Ruixin Biotechnology Co., Ltd. (Fujian province, China). ECL detection kit was purchased from GBCBIO Technologies Inc. (Guangzhou, China). XenoLight D-luciferin potassium salt was purchased from PerkinElmer (PKI, MA, USA). Hematoxylin&Eosin (H&E) staining solution was purchased from Beijing Labgic Technology Co., Ltd. (Biosharp, Beijing, China).

### Cell culture

CRC cell lines (LOVO, HCT-116, HT29 and CT26), normal intestinal epithelium cell line NCM460, and the mouse hepatic stellate cell line JS1 were purchased from Shanghai EK-Bioscience Biotechnology Co., Ltd. (Shanghai, China). Human hepatic stellate cell line LX-2 was purchased from Shanghai Guan & Dao Biological Engineering CO., Ltd. (Shanghai, China). Luciferase-expressing CT26 cells (CT26-luc) were purchased from Smart (Guangzhou) Biotechnology Co., Ltd. (Guangzhou, China).

LOVO, HT29, LX-2 and JS1 cells were cultured in high-glucose Dulbecco’s modified Eagle’s medium (DMEM) containing 10% fetal bovine serum (FBS) (Gibco, USA) and 1% penicillin/streptomycin (P/S) (HyClone, USA). HCT-116, CT26, NCM460 and CT26-luc were cultured in RPMI 1640 containing 10% FBS (Gibco, USA) and 1% penicillin/streptomycin (P/S) (HyClone, USA). Cells were cultured at 37 °C in a humidified incubator containing 5% CO_2_.

### Cell viability assay

The cytotoxic effects of brevilin A on NCM460, CRC cells and HSCs were evaluated by using the MTT assay. Cells were seeded in a 96-well plate, and then treated with various concentrations of brevilin A. After 24 h, 20 μL of MTT solution (5 mg/mL) was added and incubated for another 4 h at 37 °C. The medium was then removed, and 200 μL of DMSO was added per well. Finally, the absorbance was recorded using a microplate reader at 490 nm (BioTek, USA).

### Conditioned medium (CM) system

CRC (LOVO, HCT-116 or CT26) cells were seeded in 6-well plate cultured in RPMI 1640 containing 10% FBS for 24 h. Then, cells were washed twice with 1 × PBS and then cultured in FBS-free DMEM medium for 24 h. After that, medium was collected and centrifuged for 5 min at 1200 rpm, and then the supernatant was used to incubate with HSCs.

### Co-culture system

To explore the interaction between HSCs and CRC cells, HSCs were co-cultured with CRC cells in two-chamber dishes allowing the exchange of soluble diffusible factors while preventing their direct contact. HCT-116 and CT26 cells (10 × 10^4^ cells/well) were seeded in a polycarbonate Transwell membrane with 0.40 μm pores coated with collagen (LABSELECT, Hefei, China), LX-2 and JS1 cells were seeded in the lower chambers (6-well plates), and incubated for 24 h in FBS-free DMEM medium. Brevilin A (2.5, 5 and 10 μM) solution or IL6 neutralizing antibody (1 μg/mL) was added to the co-culture system for another 24 h. CRC cells and the supernatant were collected for detection.

### Luminex liquid suspension chip detection

Luminex liquid suspension chip detection was performed by Wayen Biotechnologies (Shanghai, China). The supernatant of CM-treated cells were collected and prepared for Luminex liquid suspension chip detection. The Bio-Plex Pro Human Chemokine Panel 40-plex kit was used in accordance with the manufacturer’s instructions.

### Cell migration assay

Cell migration was determined with 24-well Transwell chambers (8 μm pores, Corning, USA). HCT-116 or CT26 cells were seeded in the upper chamber, LX-2 or JS1 cells were seeded in the lower chambers. DMEM of 800 μL with 10% FBS was added into the lower chamber, and 200 μL DMEM with no FBS was added into the upper chamber. Invaded or migrated cells were counted and imaged by a light microscope (Leica, Germany).

### Enzyme-linked immunosorbent assay (ELISA)

Cell culture medium was collected at the end of experiments for the detection of IL6, IL8 and VEGF according to the manufacturer's instructions. Liver constitution homogenates of mouse were collected for the detection of IL6 and VEGF.

### Western blotting

Cell or tissue extracts were prepared for the Western blotting. Briefly, cell or tissue samples were collected and lysed, after that, the lysates were separated by sodium dodecyl sulfate-polyacrylamide gel electrophoresis (SDS-PAGE), transferred to polyvinylidene fluoride (PVDF) membranes, and blocked with 5% skimmed milk dissolved in tris-buffered saline tween-20 (TBST) for 1.5 h. Then, the membranes were incubated with the respective primary antibodies anti-FAP (1:1000), anti-a-SMA (1:1000), anti-VEGF (1:1000), anti-p-STAT3 (1:1000), anti-STAT3 (1:1000), anti-p-SRC (1:1000), anti-SRC (1:1000), anti-p-JAK2 (1:1000), anti-JAK2 (1:1000), or anti-GAPDH (1:1000) and secondary antibodies (goat anti rabbit IgG HRP, 1:3000; goat anti mouse IgG HRP, 1:10,000). Immunoreactive bands were visualized using an ECL detection kit (GBCBIO Technologies, China).

### Animal experiments

Male 6-week-old BALB/c mice were purchased from the Laboratory Animal Center of Southern Medical University [SCXK(GZ)2019-0041, Guangzhou, China]. They were kept in the animal laboratory at International Institute for Translational Chinese Medicine [SYXK (GZ) 2019-0144].

#### For the xenograft mouse model

The xenograft mouse model was established by subcutaneously injecting of CT26 cells (2 × 10^5^ cells/100 μL) to the BALB/c mouse. Mice were than randomly divided into 3 groups (6 mice in each group): model, brevilin A (4 mg/kg), brevilin A (8 mg/kg). Mice in model group were treated with an equivalent volume of vehicle (5% DMSO + 30% PEG-400 + 5% Tween-80 solution), others were treated with different dosages of brevilin A. All mice were treated by intraperitoneal injection every day for 2 consecutive weeks. Tumor volumes and body weights of the mice were measured and recorded every day. At the end of the experimental period, tumors of the mice were dissected and weighed. Part of the tumor tissues were frozen at − 80 °C for subsequent studies.

#### For the liver metastasis mouse model

BALB/c mice were injected intrasplenically with 2 × 10^5^ cells/100 μL CT26-luc cells under isoflurane anesthesia. Mice were randomly divided into 3 groups immediately after the cancer cell injection: model, brevilin A (4 mg/kg), brevilin A (8 mg/kg). All mice were treated as described in the above section. In order to observe the cancer metastases formation and tumor growth, mice were received an intraperitoneal injection with 150 mg/kg XenoLight D-Luciferin potassium salt twice a week and were imaged using the IVIS Lumina XRMS Series III (PerkinElmer, MA, USA). Image analyses were performed using the Living Image software 4.4.

### H&E staining and immunohistochemistry staining

The resection specimens were fixed in 10% buffered formalin and paraffin-embedded by routine processing. Sections were cut at a thickness of 4 μm, heated at 60 °C for 30 min, and then deparaffinized and hydrated through a series of xylene and alcohol baths. H&E staining was performed, followed by a dehydrating process. Histopathological examination was performed under a Zeiss microscope. Slides after deparaffinized and rehydrated were microwaved in antigen retrieval solution (citrate buffer, pH 6.0, containing 0.3% trisodium citrate and 0.04% citric acid) for 5 min. After replenishment of this solution, the slides were microwaved again for 5 min, and then allowed to cool down for 20 min. The sections were then rinsed in PBS and immersed in 3% H_2_O_2_ for 15 min to block the endogenous peroxidase activity. Thereafter, the sections were incubated with 10% bovine serum albumin (BSA) at room temperature for 1 h to block the non-specific binding. Immunohistochemical staining was performed using anti-Ki67 (1:200) antibody or anti-cleaved-caspase 3 (1:200) antibody at 4 °C for overnight. After incubating with the corresponding secondary antibodies for 2 h, the bound complexes were visualized using a SuperPicture Polymer Detection kit.

### Statistical analysis

All data are presented as mean ± SD. Statistical significance was determined by one-way ANOVA followed by Dunnett’s multiple comparisons using GraphPad Prism version 7.0 (GraphPad software, San Diego, CA, USA). *P* < 0.05 was considered statistically significant.

## Results

### CRC promotes HSCs-to-CAFs differentiation by releasing VEGF

**Fig. 1:**
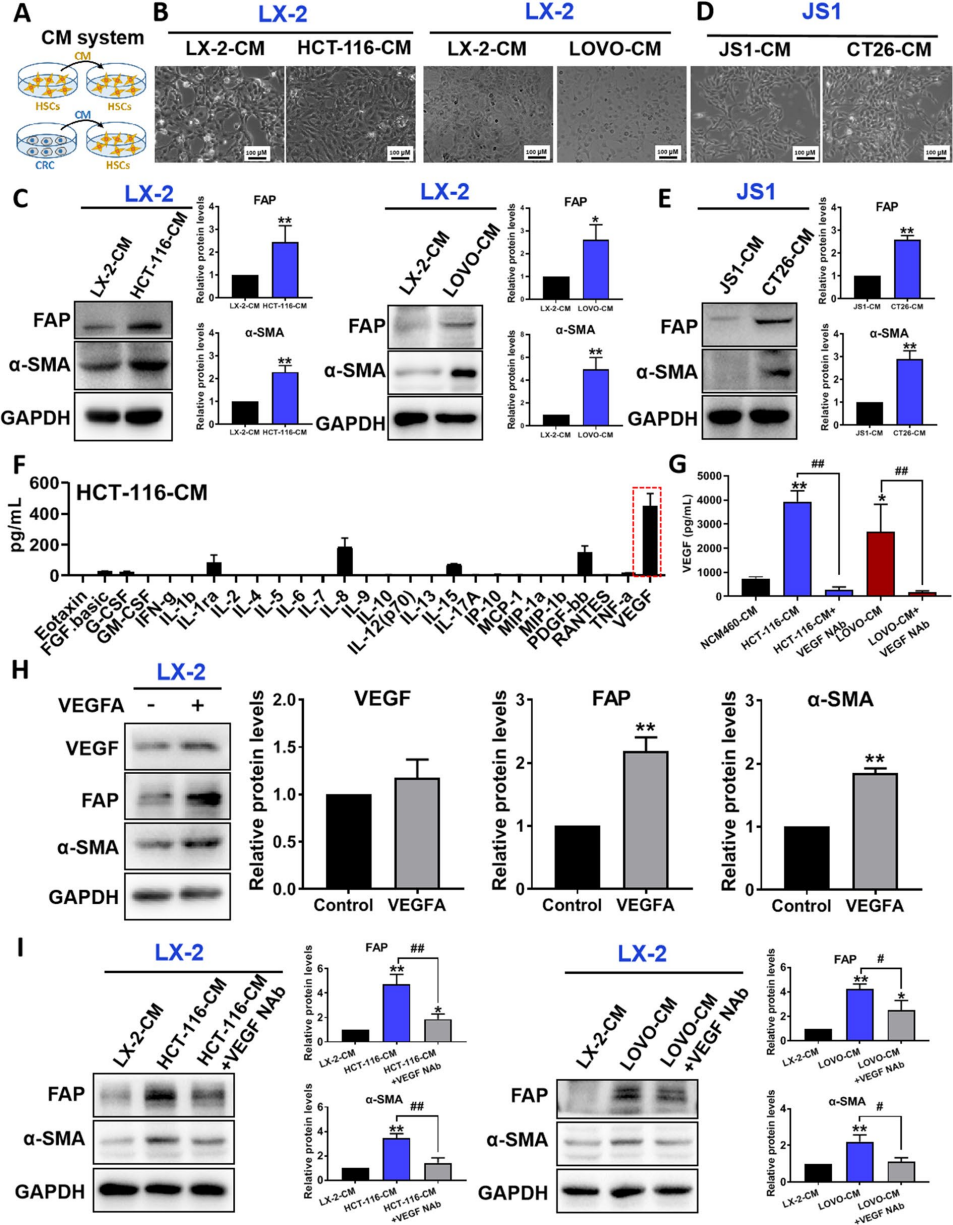
CRC promoted HSCs-to-CAFs differentiation by releasing VEGF. **A** Figure demonstrates the experimental set up for control (upper panel) and experiment (lower panel) for the conditioned medium (CM) system. **B** Cell morphology of LX-2 after treated with LX-2-CM, HCT-116-CM or LOVO-CM medium. **C** Effects of HCT-116-CM or LOVO-CM on the protein levels of FAP and α-SMA. Protein levels were examined using immunoblotting (left panel); and quantitative results were analyzed using Image J software (right panel). **D** Cell morphology of JS1 after treated with JS1-cultured medium or CT26-cultured medium. **E** Effects of CT26-CM on the protein levels of FAP and α-SMA. Protein levels were examined using immunoblotting (left panel); and quantitative results were analyzed using Image J software (right panel). **F** The levels of the cytokines or growth factors in the HCT-116 conditioned medium were detected by using the Luminex liquid suspension chip assay. **G** The VEGF contents in the medium of NCM460, HCT-116 or LOVO cells were detected by using the ELISA assay, and the VEGF contents in the medium of HCT-116 or LOVO cells treated with neutrakine VEGF165/121 monoclonal antibody were also detected by using ELISA assay. **H** Effects of VEGFA on the protein levels of VEGF, FAP and α-SMA. Protein levels were examined using immunoblotting (left panel); and quantitative results were analyzed using Image J software (right panel). **I** Effects of HCT-116-CM and HCT-116-CM + VEGF neutralizing antibody on the protein levels of FAP and α-SMA. **J** Effects of LOVO-CM and LOVO-CM + VEGF neutralizing antibody on the protein levels of FAP and α-SMA. Protein levels were examined using immunoblotting (left panel); and quantitative results were analyzed using Image J software (right panel). Data are shown as mean ± SD from three independent experiments, for **G**, **P < 0.01, *P < 0.05 *vs.* NCM460; ^##^*P* < 0.01 *vs.* CRC cells; for **C**, **E** and **H**, **P < 0.01, *P < 0.05 *vs.* the corresponding control. for **I** and **J**, **P < 0.01 *vs.* HSCs-CM, ^#^*P* < 0.05, ^##^*P* < 0.01 *vs. *CRC-CM.CM: cultured medium

We first examined whether CRC triggered HSCs-to-CAFs differentiation by treating HSCs with CRC cell conditioned medium (Fig. 1A). We found that after treating the human HSC cells (LX-2) with the CRC cells (HCT-116, LOVO) conditioned medium, the morphology of LX-2 cells were changed (Fig. 1B) and the expressions of CAF markers including alpha-smooth muscle actin (α-SMA) and fibroblast activation protein (FAP) were significantly increased (Fig. 1C). Similar results were obtained when we treated the mouse HSC cells (JS1) with the mouse CRC cells (CT26) conditioned medium (Fig. 1D, E). These data clearly showed that CRC promoted HSCs-to-CAFs differentiation.

Next, we used the Luminex liquid suspension chip assay to identify the mediators in the CRC cell conditioned medium that triggered the HSCs-to-CAFs differentiation. As shown in Fig. 1F, we found that the VEGF level was significantly higher than the other cytokines or growth factors in the conditioned medium. Subsequent ELISA study also suggests that HCT-116 cells and LOVO cells secret more VEGF than normal colon epithelial cells (NCM460), and the VEGF levels in CRC culture medium were significantly reduced by using VEGF neutralizing antibody (Fig. 1G). More importantly, treating HSCs cells with VEGFA activated the HSCs cells and promoted HSCs-to-CAFs differentiation as demonstrated by the increased expressions of FAP and α-SMA (Fig. 1H), which is consistent with the reported study [21]. Furthermore, we also found that VEGF neutralizing antibody could significantly inhibit the elevated protein levels of FAP and α-SMA in LX-2 cells that were induced by HCT-116 or LOVO conditional cultured medium, respectively (Fig. 1I, J). Our data strongly suggest that CRC cells release VEGF that promotes HSCs-to-CAFs differentiation.

### IL6 levels are significantly increased in the CRC-HSCs co-culture system and animal model of CRC with liver metastasis

**Fig. 2:**
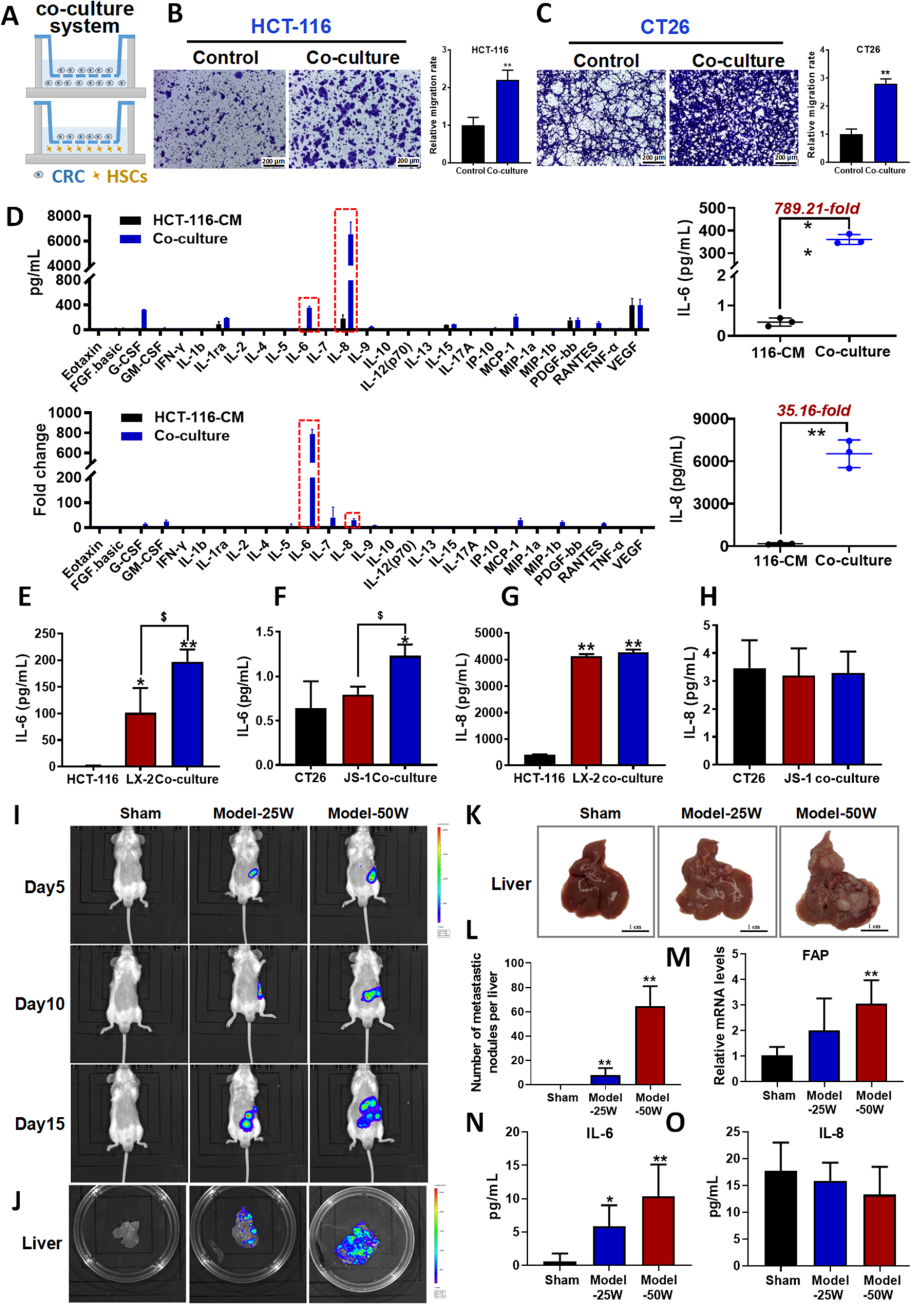
IL6 level was significantly increased in the CRC-HSCs co-culture system and animal model of CRC with liver metastasis. **A** Figure demonstrates the experimental set up for control (upper panel) and experiment (lower panel) of the co-culture system. **B**, **C** Representative photographs of HCT-116 (**B**) and CT26 (**C**) cell migration in the co-culture system (left panel); and quantitative results were analyzed using Image J software (right panel). Pictures were taken at 24 h after treatment. Data are shown as mean ± SD from three independent experiments. **P < 0.01 *vs*. the corresponding control. **D** The levels of the cytokines or growth factors in the HCT-116 conditioned medium and co-culture system were detected by using the Luminex liquid suspension chip assay. The concentration of the cytokines or growth factors in each group, and the fold change of two compared groups (left panel), as well as the levels of IL6 and IL8 (right panel) were shown. **E**, **G** The IL6 (**E**) and IL8 (**G**) content in the medium of HCT-116, LX-2 or co-culture system were detected by using the ELISA assay. **F**, **H** The IL6 (**F**) and IL8 (**H**) content in the medium of CT26, JS1 or co-culture system were detected by using the ELISA assay. **I** Luminance signals in the livers of the mice in each group. Different number of the CT26-luc cells were inoculated into the spleen of BALB/c mice. Mice were randomly divided into 3 groups, including sham-operated control, model-25w cell number and model-50w cell number groups, and the luminance signals in the livers were detected by using the IVIS Lumina XRMS Series III every 5 days. **J** Luminance signals in the liver tissues. **K** images of livers with CRC tumors. **L** Tumor number of mice. **M** The mRNA level of FAP. **N**, **O** The IL6 (**N**) and IL8 (**O**) contents in the liver tissues of the mice in each group. The homogenate of the liver tissues in each group was detected by using the ELISA assay. For **E**–**H**: data are shown as mean ± SD from three independent experiments. **P < 0.01, *P < 0.05 *vs.* CRC cells; ^$^*P* < 0.05 *vs. *HSCs. For **L**–**O**: **P < 0.01, *P < 0.05 *vs.* sham-operated control, n = 6

To further explore the interplay between CRC cells and HSCs, we co-cultured CRC cells and HSCs (Fig. 2A), and examined whether the metastatic potential of CRC were changed. Interestingly, we found that CRC cell migration was elevated in the co-culture system (Fig. 2B, C), implying some mediators in the co-culture system promoted CRC migration. Therefore, we compared the cytokine and growth factor profiles of the CRC cell conditioned medium and the CRC-HSCs co-culture conditioned medium. The data showed that both interleukin 8 (IL8) and IL6 levels were significantly elevated in the co-culture conditioned medium when compared to the HCT-116 culture alone (Fig. 2D).

Next, we determined whether IL8 and IL6 were released by CRC cells or HSCs in the co-culture system by examining their levels in the respective conditioned medium. We found that LX-2 but not HCT-116 cells released IL6 (Fig. 2E). Interestingly, the IL6 released from LX-2 was further enhanced in the presence of HCT-116 cells (Fig. 2E), suggesting that CRC cells trigger HSCs to release IL6. In the mouse cell lines, although both JS1 and CT26 released IL6, the IL6 level was significantly elevated in the co-culture conditioned medium (Fig. 2F). On the contrary, we found that HSCs released substantial levels of IL8 that were comparable to the IL8 levels in the co-culture system (Fig. 2G, H), this may explain the higher IL8 level in the co-culture conditioned medium when compared to the HCT-116 culture alone (Fig. 2D).

Further, we confirmed the above in vitro findings in animal models. We inoculated different number of CT26-luc cells into the spleen of BALB/c mice to establish the animal model of CRC with liver metastasis. Compared with sham-operated control, we found that with the increased number of injected CT26-luc cells, the fluorescence signal in the liver was enhanced (Fig. 2I, J), and the number of metastatic nodes in the liver was increased (Fig. 2K, L). Moreover, the mRNA level of FAP, a CAF marker was significantly increased when compared to the sham-operated control (Fig. 2M). More importantly, we found that with the exacerbation of liver metastases from CRC, the level of IL6 (Fig. 2N) but not IL8 (Fig. 2O) was significantly increased. Taken together, we suggest that CRC cells trigger HSCs to increase IL6 secretion but not IL8 under the co-culture conditions and in the CRC with liver metastasis.

### In the CRC-HSCs co-culture system, HSCs release IL6 that activates STAT3 in the CRC cells and increases the cancer cell migration

**Fig. 3:**
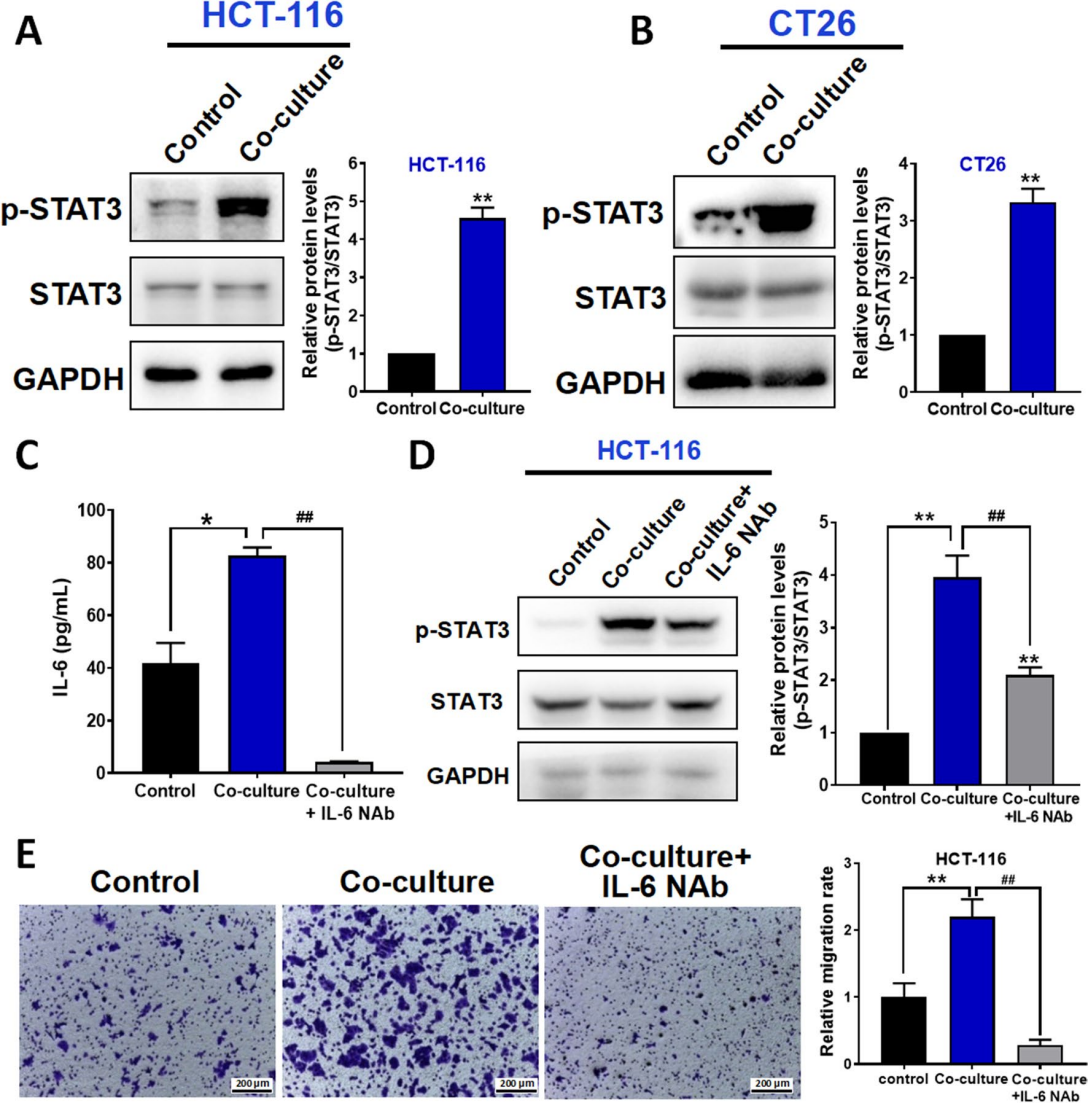
HSCs released IL6 that activated STAT3 in the CRC cells and increased cancer cells migration in the CRC-HSCs co-culture system. **A**, **B** Protein levels of p-STAT3 and STAT3 in HCT-116 (**A**), CT26 (**B**) cells and co-culture system were determined by using Western blotting (left panel); and quantitative results were analyzed using Image J software (right panel). **C** The IL6 content in the medium of HCT-116, HCT-116-LX-2 co-culture or IL6 neutralizing antibody-treated co-culture system were detected by using the ELISA assay. **D** Protein levels of p-STAT3 and STAT3 in HCT-116, HCT-116-LX-2 co-culture or IL6 neutralizing antibody-treated co-culture system were determined by using Western blotting (left panel); and quantitative results were analyzed using Image J software (right panel). **E** Representative photographs of cell migration in HCT-116, HCT-116-LX-2 co-culture or IL6 neutralizing antibody-treated co-culture system (left panel); and quantitative results were analyzed using Image J software (right panel). Data are shown as mean ± SD from three independent experiments. **P < 0.01, *P < 0.05 *vs.* the corresponding control. ^##^*P* < 0.01 *vs.* co-culture system. NAb: neutralizing antibody

Since signal transducer and activator of transcription 3 (STAT3) is a downstream target molecule of IL6 [22], the IL6 released from HSCs should activate STAT3. As shown in Fig. 3A, B, STAT3 activity in the CRC cells was significantly increased in the co-culture system. Our data suggest that CRC cells trigger HSCs to increase IL6 secretion, which in turns activates STAT3 in the CRC cells in a positive feedback manner. Indeed, with IL6 neutralizing antibody (Fig. 3C), the elevated STAT3 activity in the CRC cells in the co-culture system was significantly reduced (Fig. 3D). STAT3 has been implicated in CRC invasive ability [23], we also found that CRC cell migration was increased in the co-culture system that was reversed in the presence of IL6 neutralizing antibody (Fig. 3E). Our data clearly demonstrated the interplay between CRC cells and HSCs via the VEGF-IL6-STAT3 axis. The axis not only promotes HSCs-to-CAFs differentiation but also increases CRC cell migration.

### Brevilin A targets the VEGF-IL6-STAT3 axis in the CRC-HSCs interplay

Next, we screened for herbal compound that targeted the VEGF-IL6-STAT3 axis in the CRC-HSCs interplay. It is known that brevilin A is a STAT3 inhibitor [24]. Therefore, we investigated whether brevilin A interrupted the CRC-HSCs interplay by inhibiting the STAT3 signaling pathway.

**Fig. 4:**
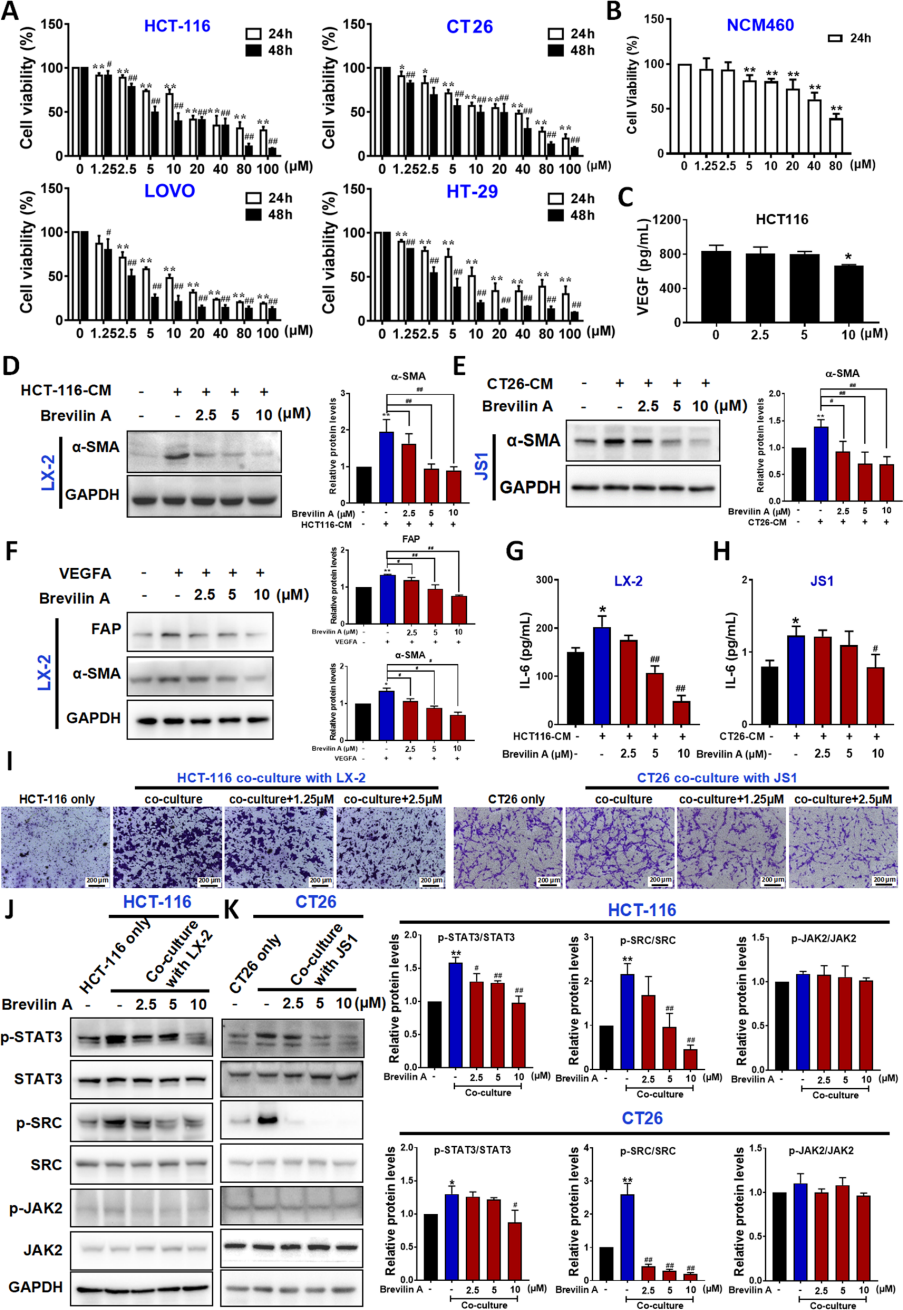
Brevilin A targets the VEGF-IL6-STAT3 axis in the CRC-HSCs interplay. **A**, **B** Brevilin A decreased viability in CRC (**A**) and NCM460 (**B**) cells. Cell viabilities were determined by the MTT assay. Data are shown as mean ± SD from three independent experiments, *P < 0.05, **P < 0.01 *vs.* vehicle (24 h); ^#^*P* < 0.05, ^##^*P* < 0.01 *vs.* vehicle (48 h). **C** The VEGF content in the medium of HCT-116 after treated with brevilin A were detected by using the ELISA assay. **D**, **E, F** Effects of brevilin A on protein levels of FAP and α-SMA in HCT-116-CM treated LX-2 cells (**D**), CT26-CM treated JS1 cells (**E**), or VEGFA-treated LX-2 cells (**F**) were determined by using Western blotting (left panel); and quantitative results were analyzed using Image J software (right panel). **G**, **H** The IL6 content in the medium of HCT-116-CM cultured LX-2 cells (**G**) or CT26-CM cultured JS1 cells (**H**) after treated with brevilin A were detected by using the ELISA assay. **I** Representative photographs of cell migration in HCT-116, HCT-116-LX-2 co-culture or brevilin A (1.25 μM and 2.5 μM)-treated co-culture system. **J**, **K** Effects of brevilin A on protein levels of STAT3, p-STAT3 (Tyr705), JAK2, p-JAK2 (Tyr1007/1008), SRC and p-SRC (Tyr416) in co-culture system were determined by using Western blotting (left panel); and quantitative results were analyzed using Image J software (right panel). Data are shown as mean ± SD from three independent expriments. For **D, E, G, H**: ^**^P < 0.01, ^*^P < 0.05 *vs. *HSCs; ^##^*P* < 0.01, ^#^*P* < 0.05 *vs.* CRC-CM treated HSCs. For **F**: ^**^P < 0.01, ^*^P < 0.05 *vs.* HSCs. ^##^*P* < 0.01, ^*#*^*P* < 0.05 *vs.* VEGFA-treated HSCs. For **J, K**: ^**^P < 0.01, ^*^P＜0.05 *vs.* CRC cells only; ^##^*P* < 0.01, ^#^*P* < 0.05 *vs. *co-culture with HSCs

Figure 4A showed that brevilin A reduced CRC cell viability in a dose-dependent manner. The IC50 values of brevilin A in HCT-116, CT26, LOVO and HT-29 cell lines were 20.98, 21.53, 8.73 and 16.70 μM, respectively after24-h treatment, and 8.94, 9.67, 2.82 and 3.05 μM, respectively after 48-h treatment. However, the IC50 for NCM460 was 57.24 μM after 24-h treatment (Fig. 4B), suggesting that brevilin A exhibited a relatively less cytotoxicity to colon epithelial cells when compared to CRC cells. More importantly, brevilin A at sub-IC50 concentrations significantly reduced the VEGF release from HCT-116 cells (Fig. 4C). Since our data and other studies [21] found that VEGF activates HSCs, the reduced VEGF secretion would affect the HSCs-to-CAFs differentiation. Indeed, brevilin A at sub-IC50 concentrations abolished the activation of HSCs that were cultured in CRC cell conditioned medium (Fig. 4D, E), or in the presence of VEGFA (Fig. 4F). We suggest that brevilin A inhibits HSCs activation, at least in part, by reducing the VEGF released by CRC cells. Besides releasing VEGF, CRC cells also triggered HSCs to release IL6 that in turns activated STAT3 in the CRC cells. Interestingly, brevilin A significantly reduced the IL6 released from HSCs that were cultured in the CRC cell conditioned medium (Fig. 4G, H). Brevilin A also significantly reduced the cell migration ability of CRC cells that were co-cultured with HSCs (Fig. 4I). SRC and JAK2 are upstream tyrosine kinases of STAT3. Here, we found that brevilin A also inhibited STAT3 signaling pathway in the CRC cells in the co-culture system as indicated by the reduced phosphorylation of STAT3, and the phosphorylation of protein kinases involved in the signaling cascade such as SRC kinase, but not JAK2 (Fig. 4J, K). Our data strongly suggest that brevilin A inhibits HSCs-to-CAFs differentiation and STAT3 signaling pathway in CRC cells by targeting the VEGF-IL6-STAT3 axis.

### Brevilin A significantly inhibits colorectal liver metastasis in vivo by targeting the VEGF-IL6-STAT3 axis

**Fig. 5:**
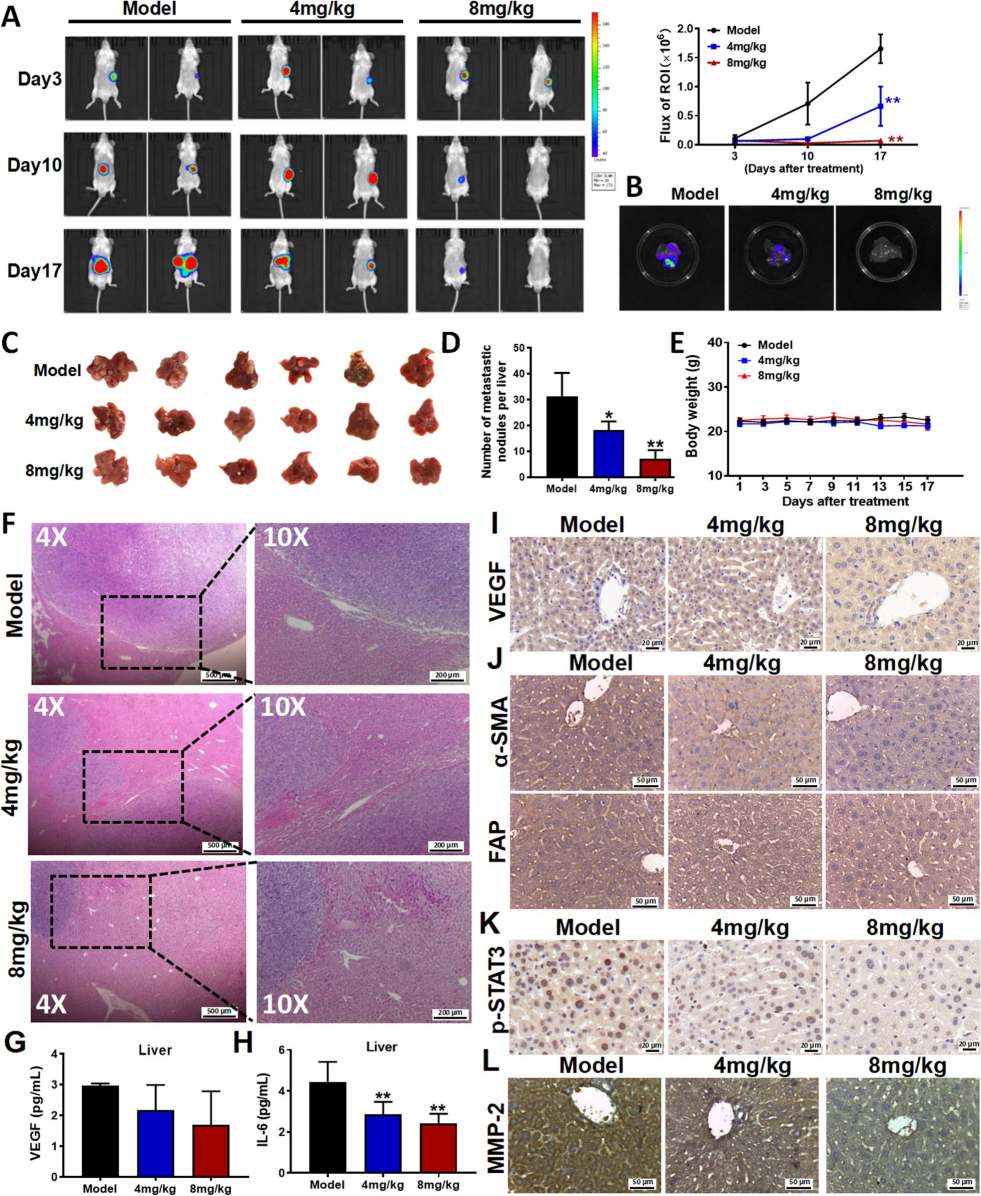
Brevilin A significantly inhibits colorectal liver metastasis by targeting the VEGF-IL6-STAT3 axis. **A** Brevilin A significantly inhibited colorectal liver metastasis in both time- and dose-dependent manners; and quantitative results were shown in the (lower panel). CT26-luc cells were inoculated into the spleen of BALB/c mice. Mice were randomly divided into model, low dosage (4 mg/kg) and high dosage of brevilin A (8 mg/kg) groups. **B** Brevilin A reduced luminance signals in the livers. **C** Images of livers with CRC tumors. **D**, **E** Tumor number (**D**) and body weights (**E**) of mice. **F** H&E staining of tumors in the liver tissues of each group. **G**, **H** The VEGF (**G**) and IL6 (**H**) contents in the liver constitution homogenate of mouse after treated with brevilin A were detected by using the ELISA assay. **I**–**L** IHC staining of VEGF (**I**), α-SMA, FAP (**J**), p-STAT3 (Tyr705) (**K**) and MMP-2 (**L**) in the liver tissues of each group. Data are presented as mean ± SD, **P < 0.01, *P < 0.05 *vs.* model group, n = 6

Next, we examined whether brevilin A inhibited colorectal liver metastasis. We inoculated the CT26-luc cells into the spleen of BALB/c mice. These mice were randomly divided into model, low dosage (4 mg/kg) and high dosage  of brevilin A (8 mg/kg) groups. Our data showed that brevilin A at both dosages significantly inhibited colorectal liver metastasis as shown by the reduced luminance signals in the livers (Fig. 5A, B), the reduced liver metastatic nodes (Fig. 5C, D). It is noted that no animal was dead in all the groups; and no significant differences in clinical signs (data not shown) and body weights (Fig. 5E) in each group. H&E staining also showed that brevilin A-treated-mice had a lower degree of tumor infiltration in the livers (Fig. 5F).

To examine whether brevilin A targeted the VEGF-IL6-STAT3 axis in vivo, we first examined the VEGF levels in the mice. Our data showed that brevilin A reduced VEGF (Fig. 5G) levels and VEGF expression (Fig. 5I) in the liver tissues. The reduced VEGF level would reduce HSCs activation. Indeed, expressions of α-SMA and FAP in the liver tissues were reduced after brevilin A treatments (Fig. 5J). Besides, IL6 levels in the liver tissues were also reduced (Fig. 5H). With the reduced IL6 levels, the p-STAT3 expression (Fig. 5K) and the metastatic potential of CRC as indicated by MMP-2 expression (Fig. 5L) were also significantly reduced.

### Brevilin A significantly inhibits CRC growth in vivo

**Fig. 6:**
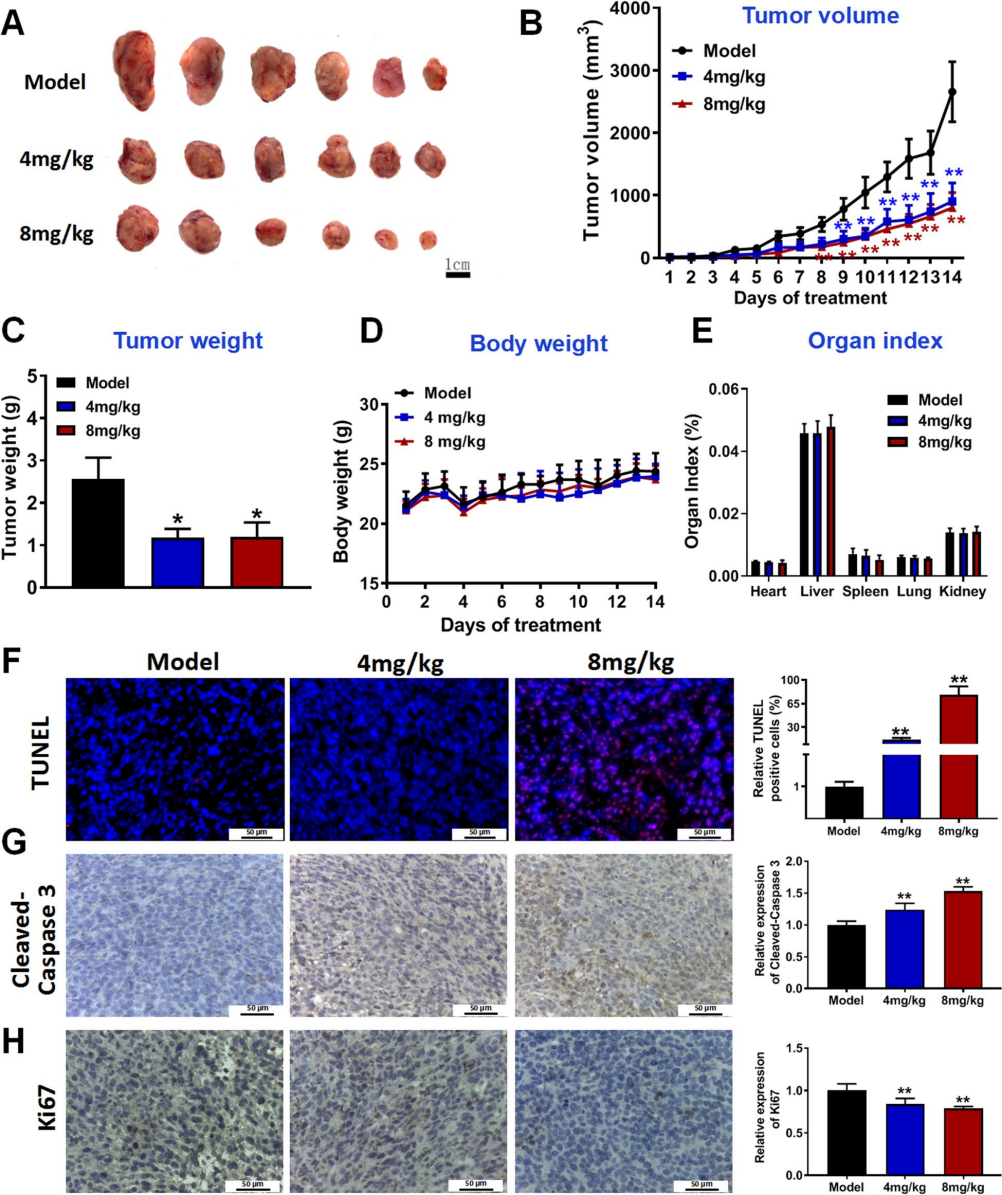
Brevilin A significantly inhibits CRC growth in vivo. **A** Representative images of tumors. **B**–**E** Tumor volume (**B**), tumor weights (**C**), body weights (**D**) and organ index (**E**) of mice. After subcutaneous inoculation of CT26 cells, mice were randomly divided into 3 groups of 6 each: vehicle (30% PEG-400/5% Tween-80 solution), 4 mg/kg of brevilin A and 8 mg/kg of brevilin A groups. **F** TUNEL staining of the tumors in each group. **G**, **H** IHC staining of cleaved-Caspase 3 (**G**) and Ki67 (**H**) in the tumor tissues; and quantitative results were shown in the (right panel). n = 6. Data are presented as mean ± SD, **P < 0.01*,* *P < 0.05 *vs. *model group

Next, we also examined whether brevilin A inhibited CRC growth because STAT3 also plays a role in cancer cell proliferation [25] and apoptosis [26]. As shown in Fig. 6A–C, brevilin A treatments at both low and high dosages significantly reduced tumor weight and volume in the CRC-bearing xenograft mouse models. The treatments did not significantly affect the body weight (Fig. 6D) and organ index (Fig. 6E) of the mice, suggesting brevilin A does not have significant toxicity to the animals. The reduced tumor growth was in part due to the enhanced apoptosis as indicated by the enhanced TUNEL staining (Fig. 6F) and the cleavage of caspase 3 (Fig. 6G) in the tumors, and the reduced cancer cell proliferation as indicated by the reduced Ki67 expressions (Fig. 6H).

## Discussion

HSCs are liver-specific pericytes that can be activated and differentiated into CAFs. CAFs are the important component in the TME because they contribute greatly to tumorigenesis [5, 11]. Identifying the mediator that the promotes the HSCs-to-CAFs differentiation and bridges the interplay between HSCs and CRC cells are the prerequisites for the development of novel therapeutic agents for mCRC treatment. Our study has clearly demonstrated the interplay between HSCs and CRC cells, in which CRC cells release VEGF that promotes HSCs-to-CAFs differentiation. CRC cells also trigger the release of IL6 from HSCs. IL6 in turns activates STAT3 signaling pathway in CRC cells and increases the cancer cell invasiveness. More importantly, we have also found that brevilin A possesses potent anti-mCRC effect by targeting the VEGF-IL6-STAT3 axis and interrupting the HSCs-CRC cell interplay.

VEGF is a well-known regulator of vascular function; it promotes angiogenesis and contributes to the pathologic condition in many cancer types. In CRC, loss of the intracrine VEGF signal increases spontaneous apoptosis and chemosensitivity [27]. VEGF receptors (VEGF-Rs) are also highly expressed in the micro-vessels of CRC [28]. Through the intracrine VEGF/VEGF-R1 signaling mechanism, VEGF can increase the metastatic potential of CRC [29]. In fact, a high VEGF-R1 expression is correlated with shorter post-operative survival in stage II/III CRC patients (*P* = 0.01) [28]. Besides, VEGF has been reported to activate HSCs. For example, a study showed that VEGF released from endothelial cells increases HSCs proliferation and promotes HSCs-to-CAFs differentiation [21]. Besides, VEGF released from hepatocytes activates HSCs and leading to non-alcoholic fatty liver disease and hepatocellular carcinoma development [30]. In this study, we found that VEGF is also released from CRC, that activates HSCs. However, we have not examined whether VEGF-R1 expression in HSCs is associated with the colorectal liver metastasis or the cancer stages. Furthermore, since other cell types such as hepatocytes or endothelial cells in the TME would also release VEGF that contributes to the cancer pathology, it is not clear about the contribution of the VEGF that is released by CRC. Nevertheless, our data suggest that the VEGF released by CRC promotes HSCs-to-CAFs differentiation that significantly enhances the colorectal liver metastasis.

Serum IL6 has been associated with CRC development. Although previous clinical studies report that CRC patients have significantly enhanced IL6 levels that correlate with tumor size, stage, metastasis and survival rate [31–35], the source of the IL6 is unknown. Serum IL6 can be released by macrophages, monocytes, fibroblasts and cancer cells [31]. Our study has clearly demonstrated that CRC triggers HSCs to increase the release of IL6, which may be a rationale underlying the elevated IL6 levels in the CRC patients.

Our findings have demonstrated the pro-metastatic role of IL6, which is also supported by other studies. A study showed that in IL6-deficient mice, the liver metastatic colonization of CRC cells is significantly reduced [36]. Besides, CRC also increases the secretion of IL6 from liver fibroblast, which in turns increases CRC invasion [37]. However, whether IL6 released by liver fibroblast will also activate HSCs remains unknown. Another study also shows that HSC conditioned medium increases CRC growth and metastasis [17], but the involving mediator has not been clearly defined, which may be platelet-derived growth factor, hepatocyte growth factor, or transforming growth factor-beta [17, 38]. Therefore, the study may not be able to fully elucidate the interplay between HSCs and CRC cells.

Based on the pro-metastatic role of IL6, targeting IL6 is a pragmatic strategy for mCRC treatment. Besides, targeting IL6 also has an added advantage because high serum IL6 level would reduce the therapeutic efficacy of the anti-VEGF antibody bevacizumab in mCRC [39]. IL6 is a pleiotropic cytokine. IL6 binds to both the membrane bound IL-6 receptor (IL-6R) and soluble IL-6R (sIL-6R). The binding of IL-6 to sIL-6R induces IL6 signaling in all the gp130-expressing cells [40]. Therefore, IL6 not only regulates the cellular immune response, but also activates a vast array of signaling pathways in different cell types in the TME that facilitate cancer development. However, inhibiting IL6 activity with neutralizing antibody may not be an ideal approach for the cancer treatment because the therapeutic efficacy not only depends on the antibody-to-cytokine binding affinity, but also the initial IL6 concentrations in the CRC patients [41], which may be a clinical challenge. Currently, a monoclonal antibody against IL6, Siltuximab, is approved by the US Food and Drug Administration for the treatment of multicentric Castleman disease [42]. However, Siltuximab monotherapy did not show efficacy against CRC patients [43]. Clinical phase II trial has been done to test the therapeutic efficacy of Siltuximab in solid cancers. With the primary efficacy endpoint set at more than 6 weeks of complete response, partial response or stable disease, only 5 out of the 84 CRC patients achieved the stable disease for more than 6 weeks [43]. Besides, the treatments also lead to adverse events, 98% of the patients reported fatigue, nausea, constipation, neutropenia, leukopenia or lymphocytopenia [43]. These clinical data suggest that IL6 antibody-based therapy cannot offer clinical benefit to the CRC patients.

Targeting the downstream signaling molecules of IL6 is feasible. OPB-31121 is a novel STAT3 inhibitor with high affinity for the SH2 domain of STAT3, it shows significant anticancer activity in preclinical studies [44]. In the Otsuka Pharmaceutical-led Phase I study, OPB31121 also showed potent antitumor activity in patients with advanced CRC [45]. Other preclinical trials have suggested a STAT3 antisense oligonucleotide AZD9150 [46] and STAT3 inhibitor bruceantinol [47] for CRC treatments. However, STAT3 is not the only downstream signaling molecule of IL6. IL6 also triggers other signaling pathways such as JAK/STAT, Ras/MAPK and PI3K/AKT and NF-κB signaling pathways [48–50]. Hence, STAT3 inhibitor may not be the ideal therapeutic agent to treat mCRC because they cannot interrupt the interplay between HSCs and CRC cells which is mediated by VEGF and IL6 as demonstrated in our study.

**Fig. 7:**
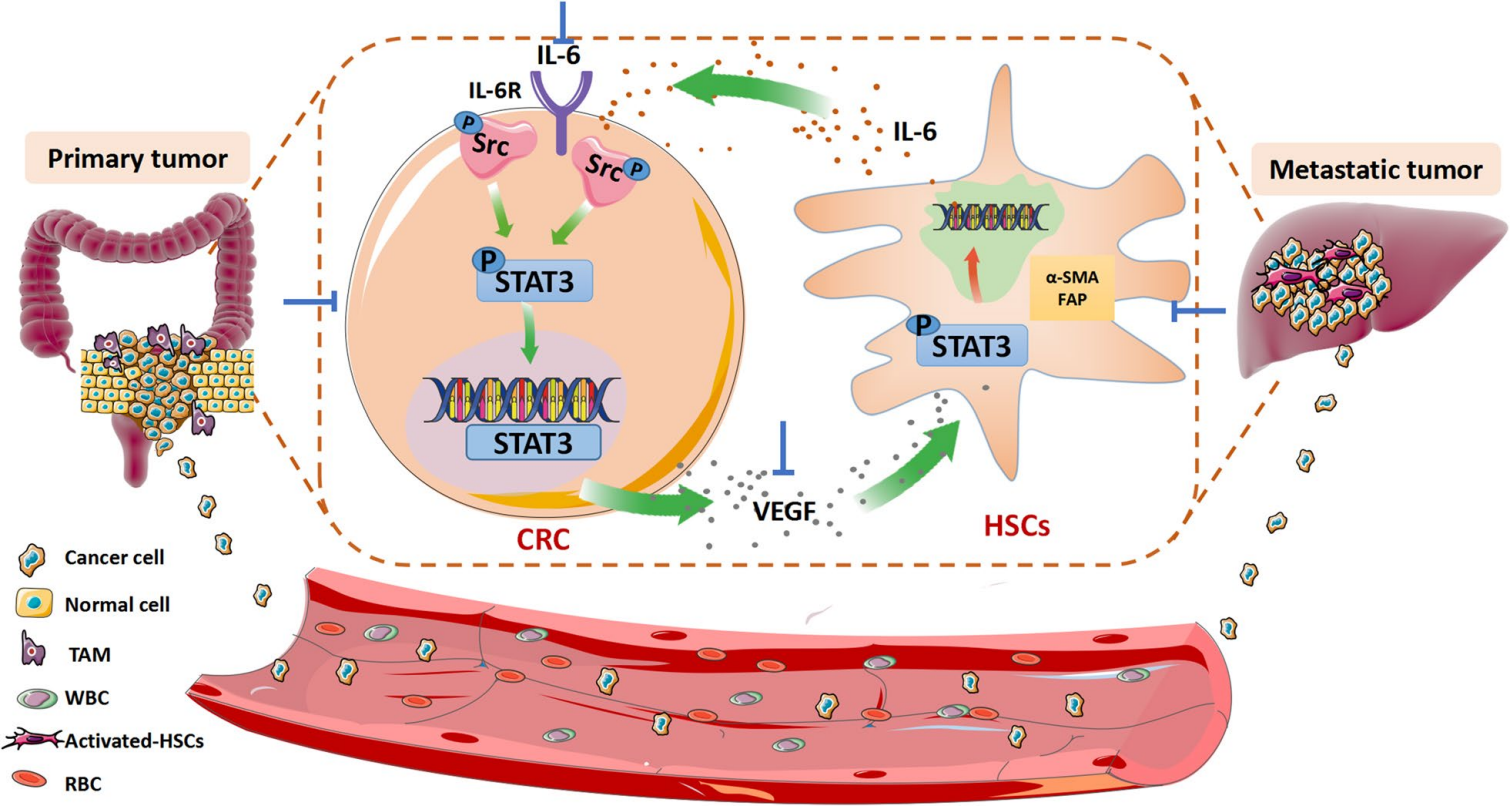
A schematic diagram showing the roles of VEGF-IL6-STAT3 axis in the HSCs-CRC interplay; and the mechanism underlying the inhibitory effect of brevilin A on CRC liver metastasis

To develop effective therapeutic strategy, we have to understand the interplay between CRC and HSCs. In this study, we demonstrated that CRC cells trigger HSCs to increase IL6 secretion, which in turns activates STAT3 in the CRC cells in a positive feedback manner. Our further study showed that there was no significant effects on the VEGF release and protein expression levels with IL6 neutralizing antibody, suggesting that IL6 does not modulate VEGF (Additional file 1: Fig. S1), it is mainly released through HSCs and then promotes STAT3 activation in CRC. We are the first to demonstrate the role of VEGF-IL6-STAT3 axis in mediating the CRC-HSCs interplay, in which activation of this axis promotes HSCs-to-CAFs differentiation and colorectal liver metastasis (Fig. 7). Our findings have suggested a novel therapeutic target for the treatment of mCRC. Besides, we are also the first to reveal the role of brevilin A in targeting the VEGF-IL6-STAT3 axis in the HSCs-CRC interplay. In the co-culture system, brevilin A abolishes the HSCs-to-CAFs differentiation, inhibits the cancer STAT3 activity and reduces the cancer metastatic potential. Our exciting data also showed that brevilin A at 8 mg/kg completely inhibits colorectal liver metastasis in mouse models.

Brevilin A is a natural sesquiterpene lactone. A number of brevilin A derivates have been synthesized and their anti-cancer effects have been evaluated in structure–activity relationship studies [51]. Although some of the structural modifications such as introducing different substituents to the alpha-position of the gamma-lactone ring enhance the anti-cancer effects of the brevilin A synthetic compounds [20], whether these synthetic or semi-synthetic compounds would also target the VEGF-IL6-STAT3 axis in the HSCs-CRC interplay has not been studied. Furthermore, the toxicities of these synthetic compounds may also hinder the clinical translation into therapeutic agents for mCRC treatments. Furthermore, same as many FDA approved drugs, brevilin A contains Michael acceptor. Many natural compounds also contain Michael acceptor and exert a vast array of therapeutic functions [52]. The covalent binding of target proteins using Michael acceptor moieties has been shown to improve pharmacodynamic properties of the drugs and prolong the duration of the pharmacological effects [52]. Brevilin A should have a great translational potential to be developed as therapeutic agents for disease treatments.

## Conclusions

Our data have clearly demonstrated the role of the VEGF-IL6-STAT3 axis in mediating HSCs-CRC interplay, in which CRC releases VEGF that triggers HSCs-to-CAFs differentiation. CRC also enhances the release of IL6 from HSCs, which in turn activates STAT3 signaling pathway in the CRC cells and increases colorectal liver metastasis. More importantly, our study has also demonstrated that brevilin A significantly inhibits colorectal liver metastasis by targeting the VEGF-IL6-STAT3 axis. Our study has provided strong scientific evidence to support the clinical translation of brevilin A into a potent therapeutic agent for the treatment of mCRC.

## Supplementary Information

**Additional file 1: Figure S1.** IL6 does not modulate the VEGF release. **A** The VEGF content in the medium of HCT-116, HCT-116-LX-2 co-culture or IL6 neutralizing antibody-treated co-culture system were detected by using the ELISA assay. **B** Protein levels of VEGF in HCT-116, HCT-116-LX-2 co-culture or IL6 neutralizing antibody-treated co-culture system were determined by using Western blotting.

## Data Availability

Data collected for this study will be made available on request after article publication.

## Abbreviations

α-SMA: α-Smooth muscle actin
BSA: Bovine serum albumin
CAFs: Carcinoma-associated fibroblasts
CM: Conditioned medium
CRC: Colorectal cancer
DMSO: Dimethyl sulfoxide
DMEM: Dulbecco’s modified Eagle’s medium
ECM: Extracellular matrix
FAP: Fibroblast activation protein
FBS: Fetal bovine serum
HSCs: Hepatic stellate cells
H&E: Hematoxylin–Eosin
JAK2: Janus kinase-2
IL-6R: L-6 receptor
IL8: Interleukin 8
IL6: Interleukin 6
mCRC: Metastatic colorectal cancer
PVDF: Polyvinylidene fluoride
STAT3: Since signal transducer and activator of transcription 3
sIL-6R: Soluble IL-6R
SDF-1: Stromal cell-derived factor 1
SDS-PAGE: Sodium dodecyl sulfate-polyacrylamide gel electrophoresis
TME: Tumor microenvironment
TBST: Tris-buffered saline tween-20
TGF-β1: Transforming growth factor-beta1
VEGF-Rs: VEGF receptors
