# Supplementary figures and images for: Brevilin A is a potent anti-metastatic CRC agent that targets the VEGF-IL6-STAT3 axis in the HSCs-CRC interplay
Source: J Transl Med. 2023 Apr 16;21:260. doi: 10.1186/s12967-023-04087-6 (PMC10105967; doi:10.1186/s12967-023-04087-6)

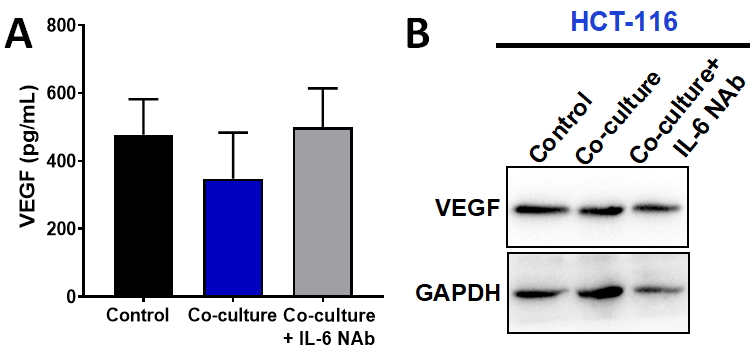

Supplement: Supplementary file 1 — Additional file 1: Figure S1. IL6 does not modulate the VEGF release. A The VEGF content in the medium of HCT-116, HCT-116-LX-2 co-culture or IL6 neutralizing antibody-treated co-culture system were detected by using the ELISA assay. B Protein levels of VEGF in HCT-116, HCT-116-LX-2 co-culture or IL6 neutralizing antibody-treated co-culture system were determined by using Western blotting. [file 12967_2023_4087_MOESM1_ESM.tif]
